# Analysis of Machine Learning-Based Assessment for Elbow Spasticity Using Inertial Sensors

**DOI:** 10.3390/s20061622

**Published:** 2020-03-14

**Authors:** Jung-Yeon Kim, Geunsu Park, Seong-A Lee, Yunyoung Nam

**Affiliations:** 1 ICT Convergence Rehabilitation Engineering Research Center, Soonchunhyang University, Asan 31538, Korea; betterwithme@sch.ac.kr; 2 Department of ICT Convergence Rehabilitation Engineering, Soonchunhyang University, Asan 31538, Korea; visionlove01@gmail.com; 3 Department of Occupational Therapy, Soonchunhyang University, Asan 31538, Korea; myanmy@sch.ac.kr; 4 Department of Computer Science and Engineering, Soonchunhyang University, Asan 31538, Korea

**Keywords:** spasticity assessment, machine learning, wearable sensor technologies, inertial measurement unit, rehabilitation engineering, tele-rehabilitation

## Abstract

Spasticity is a frequently observed symptom in patients with neurological impairments. Spastic movements of their upper and lower limbs are periodically measured to evaluate functional outcomes of physical rehabilitation, and they are quantified by clinical outcome measures such as the modified Ashworth scale (MAS). This study proposes a method to determine the severity of elbow spasticity, by analyzing the acceleration and rotation attributes collected from the elbow of the affected side of patients and machine-learning algorithms to classify the degree of spastic movement; this approach is comparable to assigning an MAS score. We collected inertial data from participants using a wearable device incorporating inertial measurement units during a passive stretch test. Machine-learning algorithms—including decision tree, random forests (RFs), support vector machine, linear discriminant analysis, and multilayer perceptrons—were evaluated in combinations of two segmentation techniques and feature sets. A RF performed well, achieving up to 95.4% accuracy. This work not only successfully demonstrates how wearable technology and machine learning can be used to generate a clinically meaningful index but also offers rehabilitation patients an opportunity to monitor the degree of spasticity, even in nonhealthcare institutions where the help of clinical professionals is unavailable.

## 1. Background

Spasticity is a symptom of neurological impairment and is prevalent in patients with stroke [1,2], multiple sclerosis [3], cerebral palsy [4], or spinal cord injury (SCI) [5,6]. It is characterized by a velocity-dependent increase in muscle tone during passive stretch [7]. Spastic movement of upper or lower limbs are measured at periodical intervals to monitor patient progress. However, it remains complicated to correctly quantify spasticity, despite the application of diverse approaches from different academic fields.

Clinical professionals have come up with outcome measures to evaluate spasticity. One of the most common approaches is to apply a clinical scale, such as modified Ashworth scale (MAS) [8] and modified Tardieu scale [9]. Although such clinical constructs have been challenged in terms of their reliability, particularly inter-rater reliability [10,11,12], these measures are frequently used in practice as they are simple to carry out. In fact, the most commonly used clinical measures for spasticity are the Ashworth scale and the MAS [13].

Researchers have attempted to evaluate spasticity employing a variety of sensors to capture physiological and biomechanical signals. The recorded signals are analyzed to derive clinically meaningful indexes and to compare the results with clinical scales. McGibbon et al. [14] proposed a wearable system that consisted of a fiberoptic goniometer and an electromyography (EMG) sensor with two channels to record kinematic responses and muscle activity during passive stretch-reflex tests of spasticity under elbow flexion and extension. Associations between MAS scores and metrics extracted from kinematic and EMG data that represent the intensity of involuntary reflex have been evaluated. Pandyan et al. [15] developed a biomechanical measure of resistance to passive movement that incorporates a force transducer and a flexible electrogoniometer for use in a clinical setting for patients with different health conditions, such as traumatic brain injury, stroke, and multiple sclerosis. In addition, applied force, passive range of movement, and speed of the device have been used as indicators of elbow spasticity and compared to the MAS score. Spasticity assessment based on multimodal signals has also been applied to children with cerebral palsy [16].

Wearable sensors, such as internal sensors, are increasingly being used in rehabilitation studies that explore the possibility of inertial data to assess spasticity of both upper and lower limbs in patients with neurological disorders. Van den Noort et al. [17] introduced a method to assess the spasticity of lower limbs including the medial hamstrings, soleus, and gastrocnemius in children with cerebral palsy. They determined the angle of catch, which refers to a sudden stop or increased resistance during dynamic joint movement at a certain angle before being fully extended or flexed [18], from the inertial signals transforming the three-dimensional (3D) orientations of inertial sensors to the 3D joint angle of the lower limb. This method has also been tested on the upper limbs of stroke patients and has demonstrated excellent test-rest and inter-rater reliability [19]. A similar method utilizing inertial sensors for accurate and reliable assessment of spasticity was proposed by Choi et al. [20]; they added visual biofeedback to their inertial sensor-based spasticity assessment to provide assessors with additional information on the joint movement of lower limbs in regular passive stretch velocity to improve reliability.

Wearable sensors have been successfully demonstrated in healthcare research. Applications commonly used include continuous monitoring of activities of daily living [21,22], gait, and mobility [23,24]. Although the increasing use of wearable sensors poses great challenges to data analyses as the sensors record significant amounts of time-series data, the rapid development of data analytic methods has enabled vast amounts of data to be processed, revealing hidden information. Machine learning is a widely used data analytics technique, which uses statistical techniques to learn from the observed data and predict outcomes or categorize observations in unseen data. Many attempts have been made to investigate the efficacy of machine learning for the delivery of rehabilitation services. Yang et al. [25] developed a hand function recovery system consisting of a smart wearable armband that incorporates surface EMG to measure bio-potential signals and machine-learning algorithms to detect different hand movement patterns, and a dexterous robot hand to mimic the user’s hand gestures. They applied machine-learning algorithms to sensor data to provide interventions, as a promising technology to improve the degree of automation and the quality of intelligent decision making in healthcare service delivery.

Apart from the studies presenting the applications of machine learning as a way of providing interventions, recent evidence has demonstrated the successful use of machine learning in outcome assessments. The performance of a client during a rehabilitation exercise can be classified according to whether he or she performs the given exercise correctly [26]. For spasticity assessment, artificial neural networks are often applied to learn patterns of biomechanical data recorded by multiple sensors, including force sensors and angle sensors embedded in wearable devices [27,28]. Zhang et al. [29] used regression-based supervised learning algorithms to predict MAS scores based on EMG signals and inertial data a triaxial accelerometer, a triaxial gyroscope, and a triaxial magnetometer.

A large number of studies have been conducted to develop spasticity assessment methods using advanced technologies, with the aim of providing clinical professionals with reliable information related to characteristics of spasticity. However, there is a growth in demand for outcome measures to be implemented in home, community, and nonhealthcare institutions as patients with neurological impairments require continuous rehabilitation to maintain or improve their condition. Even when they are discharged from hospitals, it is necessary to continuously monitor the condition for appropriate rehabilitation treatment. Despite some success, the need to use extra devices to collect the data and the need for a clinician/professional to be present to assess spasticity has complicated remote monitoring of this condition in nonhealthcare facilities. Such instruments also tend to be costly. This has limited the tools available to rehabilitation clients for health monitoring. Therefore, developing low-cost and simple methods for assessing spasticity in remote nonhospital environments without the help of healthcare professionals is of great importance. To address this issue, we propose a machine-learning method to provide information regarding the degree of spasticity of an elbow using a wearable device with inertial measurement units (IMUs).

## 2. Materials and Methods

### 2.1. Participants

The study was approved by the Soonchunhyang University Institutional Review Board (no. 1040875–201909-BM-050). Recruitment for participants took place at a long-term care hospital. Patients who expressed interest in the study were considered potential participants, and they were screened to determine eligibility. Patients were excluded if their cognitive function was impaired (the mini-mental state examination score ≤ 23), they expressed discomfort in using a wearable device, or if the assigned therapist judged the participant to be unfit. After screening, the study format was explained to the patients, and informed consent was obtained prior to the experiment. Initially, 50 patients were selected. However, one patient ceased to participate, and another patient had a technical issue with the wearable device. Therefore, the reported results were obtained from 48 participants. The patients’ demographic information is summarized in Table 1.

Most of the male participants (n = 26) were admitted to the institution due to a cerebrovascular accident (CVA), with the exception of three participants with SCI. Nine of the 26 male participants had no spastic symptoms in their upper limbs. Seven male participants were affected with in their right upper limb; the rest of male participants were affected on their left side. Female participants (n = 22) were affected mostly by CVA; one female patient was admitted due to SCI. Eight female participants had no spastic movements in their upper limbs; 10 of the female participants appeared to have spastic symptoms in their right upper limb; only four female participants had the symptoms in their left upper limb.

### 2.2. Measurement of Elbow Spasticity

A frequently used assessment tool to measure the severity of spasticity is MAS [8,30]. The original Ashworth scale [31] was designed to measure spasticity in five degrees. This was modified by Bohannon and Smith [8] such that a new measurement (1 +) falls between 1 and 2. Therefore, the degree of spasticity is rated as 1 + if a slight increase in muscle tone is observed, which can be manifested by a catch, followed by minimal resistance. Table 2 describes the scoring rule as suggested by those authors.

### 2.3. Experimental Setup

In this study, we used an off-the-shelf wearable device that incorporates a three-axis accelerometer, a three-axis gyroscope, and a three-axis magnetometer (Shimmer Sensing, Dublin, Ireland). The device is equipped with a TI MSP 430 microcontroller (24 MHz, 16-bit) for processing and an RN42 Bluetooth module for wireless data transmission [32]. The wearable device was placed on the dorsal side of the affected elbow of participants. If the participant had no spastic symptoms, the device was placed on the dominant side of the elbow.

### 2.4. Data Collection

Each participant was asked to place the wearable device on the wrist of the affected side for the experiment. A rehabilitation therapist instructed him or her on the proper position for sitting in a fixed chair. Once positioned, the patient was instructed to remain relaxed and to avoid voluntary movement during the experiment. The degree of spasticity was quantified using the scoring method described in a previous study [8] when passive stretch-reflex testing of the elbow in flexion and extension was being carried out according to instructions explained in [31]. The therapist placed the participant’s forearm in a neutral position and had the elbow fully flexed, then extended the elbow from maximum possible flexion to maximum possible extension five times using one hand, while holding the elbow in the other to prevent a significant change in elbow position. The therapist moved the limb at the speed of gravity as spasticity is dependent on velocity.

**Figure 1:**
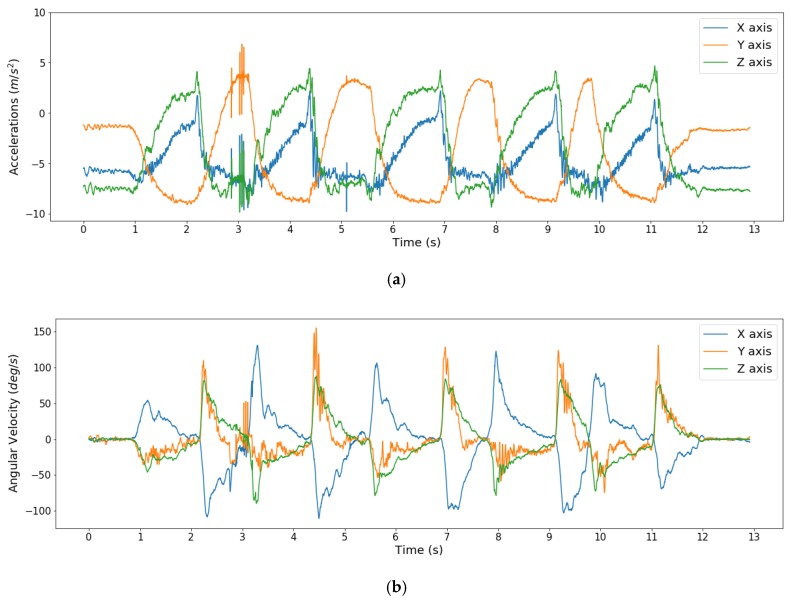
Raw tri-axial acceleration (**a**) and angular rotation (**b**) recorded from the elbow of a participant during spasticity assessment using the MAS.

Inertial data reflecting spastic movements were collected by a therapist, in which the degree of spasticity of the affected elbow was based on the MAS. The therapist held the affected arm of a participant still (quasi-static states) to stabilize signals of IMUs, then had the elbow moved by one cycle per second [20,33]. A considerable number of studies have employed machine learning on EMG and inertial data as the two sensors types are commonly used to assess spasticity. Although various sampling rates have been used depending on the movement type, activities with fast movement have been sampled at a relatively higher sampling frequency, whereas lower sampling frequencies have been used for the activities with a slower movement. EMG readings tend to be sampled at relatively high sampling frequency (1 KHz) [14,15], whereas inertial signals are sampled at lower frequencies ranging from 100 Hz to 204.8 Hz [20,29]. In this study, inertial data were sampled at 256 Hz during the experiments, to avoid missing valuable information. An example of the inertial signals recorded during the experiment is shown in Figure 1.

### 2.5. Signal Preprocessing

**Figure 2:**
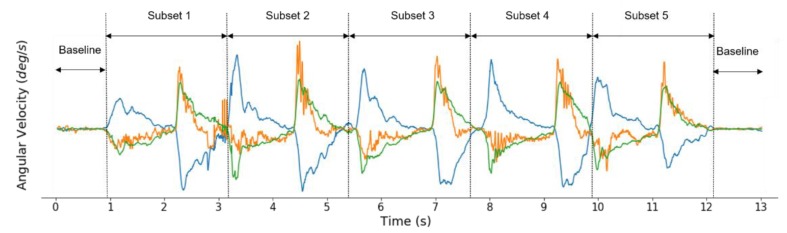
Baseline recordings and subsets of elbow flexion–extension movement cycles.

Prior to feature computation, raw signals were preprocessed to obtain inertial signals that represented the spastic movements of the elbow of a participant. First, baseline recordings of inertial data collected during spasticity assessment were determined by the angular velocity and discarded. This step ensured acquisition of some inertial data of elbow movements. To obtain the most representative acceleration and angular velocity, the inertial signals were separated to select the middle three cycles of elbow flexion and extension from the total of five cycles. This was achieved by first dividing the signals into five subsets and then removing the first and last subsets. The parts of the signals are described in Figure 2. These steps resulted in inertial signals reflecting only the three cycles of elbow flexion and extension.

**Figure 3:**
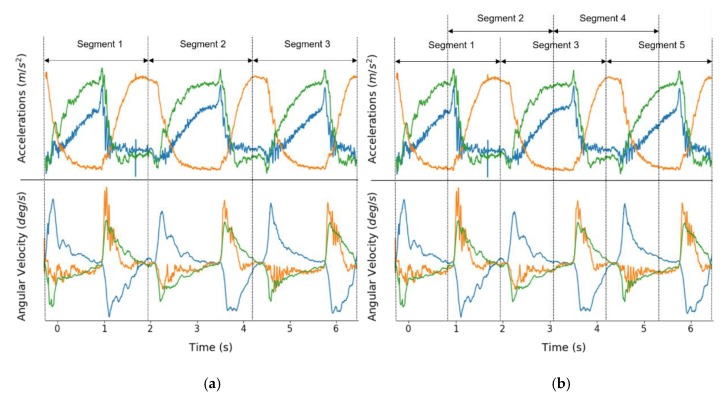
Two different segmentation schemes tested in this study: (**a**) nonoverlapping segmentation; (**b**) segmentation with 50% overlapping.

After obtaining the portion of the inertial signal containing the most stable cycles of elbow movement, segmentation was used to divide the signals into subgroups of signals that shared common characteristics of elbow flexion and extension movements, according to the MAS score. Two segmentation techniques were applied. Figure 3 illustrates the two techniques using the part of inertial signals that reflected the three cycles of elbow flexion and extension.

One of them was to segment signals without overlap, whereas the other method allowed 50% overlap, which means that a segment was created with half of the data samples from the previous window and the other half from the next window. There was no consensus in overlapping percentage; 50% overlap was tested as it had been widely accepted in related studies [34,35,36]. Two extra segments were produced from the same inertial data. The former produced three segments of each signal leading to three segments containing triaxial acceleration and angular rotation per participant (6 × 3). The latter created five segments per participant (6 × 5). Through the preprocessing stage, two datasets were obtained: dataset 1 (DS1) and dataset 2 (DS2).

### 2.6. Feature Extraction

After the preprocessing phase, features were computed from each segment to classify severity of spasticity. Two feature sets were prepared to investigate the impact of feature types on classification performance. The most common statistical features—e.g., root mean square, mean, standard deviation, energy, spectral energy, absolute difference, and variance [37]—were extracted from the datasets, resulting in 42 features referred to as feature set 1 (FS1). Furthermore, additional features were computed to create another feature set according to the steps given below. First, acceleration and angular velocity data were converted into rotation values, such as roll and pitch, using Equations (1) and (2):

(1) $$\mathrm{roll}=\frac{180}{\Pi }tan^{-1}\left(\frac{y}{g},\frac{z}{g}\right),$$

(2) $$\mathrm{pitch}=\frac{180}{\Pi }tan^{-1}\left(\frac{x}{g},\frac{z}{g}\right),$$

where x, y, and z represent accelerations in the x, y, and z directions, and g refers to the acceleration due to gravity. Common statistical features were computed based on these data. In addition, two extra features, signal magnitude area (SMA) and signal vector magnitude (SV) were derived from accelerations in the x and y directions. SMA indicates periods of activity and rest and SV reflects the degree of movement intensity. SMA and SV were computed using Equations (3) and (4) as [38]:

(3) $$\mathrm{SV}=\frac{\sum_{i=1}^{n}\sqrt{x_{i}^{2}+y_{i}^{2}}}{n},$$

(4) $$\mathrm{SMA}=\frac{\sum_{i=1}^{n}\left(\left|x_{i}\right|+\left|y_{i}\right|\right)}{n}.$$

The additional feature computation process extracted an extra 16 features; these were added to FS1, resulting in feature set 2 (FS2) with a total of 58 features. Table 3 summarizes the feature sets.

### 2.7. Machine-Learning Algorithms and Performance Evaluation

Machine-learning classifiers were used to automatically infer a function, which can be used to predict categories, from labeled data that were processed from inertial signals, which were collected during passive stretching. The inertial signals were labeled with the MAS scores rated by the therapist. This type of task is a supervised learning problem.

In the area of supervised learning, there are several widely used supervised learning classifiers, including linear discriminant analysis (LDA), support vector machines (SVMs), decision tree (DT), random forests (RFs), and multilayer perceptrons (MLPs). LDA is a generalization of Fisher’s linear discriminant [39], which has been widely used in statistical pattern recognition [40]. Although LDA is capable of binary classification in its original form, it can be extended to perform multiclass classification through multiple discriminant analysis as described in [41]. LDA requires continuous variables as input and produces a categorical variable as output (i.e., class label).

SVMs have shown excellent performance in classification tasks [42]. An SVM constructs a hyperplane or a set of hyperplanes, which refers to decision boundaries that has maximum margin between data points, in an N-dimensional space, where N refers to the number of features that optimally separate data points into categories. SVMs are also a binary classifier in their simplest form. However, multiclass classification can be achieved through reduction methods, such as one-versus-the-rest method [43], pairwise classifications [44], error correcting output coding [45], and direct acyclic graph [46]. Although SVMs are a type of linear classifiers, it can be used for nonlinear classification by applying kernel functions.

DTs are a commonly used in decision analysis, whereas they are also a popular method in machine learning. DTs build a model in the form of a tree structure that predicts an output variable by learning simple decision rules inferred from the features of training data. It divides a dataset into smaller subsets while an associated DT is incrementally developed by utilizing an if–then rule set at the same time. While there are certain advantages of DTs, e.g., the ease of interpretation and visualization of the result obtained, they have a few disadvantages. For example, DT models are prone to overfitting. It means small changes in the training data can result in significant changes in the structure of the optimal decision tree, which eventually leads to overall poor performance. To address the issue, random forests (RFs) were introduced [47]. RFs consist of a large number of individual decision trees as the name itself implies. RFs operate are an ensemble learning method, in which relatively uncorrelated decision trees are constructed at training phase and yield a prediction, which is the mode of the classes of each decision tree. The idea of RFs is to grow a deep tree on each subsample that perfectly predicts output for the local data (overfitting) and then apply the ensemble technique to reduce the overall variance.

A multilayer perceptron (MLP) is a feedforward artificial neural network. A simple form of MLP consists of at least three layers: an input layer, a hidden layer, and an output layer, which are fully connected in its basic form. However, MLPs can be extended to have multiple hidden layers [48]. Each node is a neuron that uses a nonlinear activation function, with the exception of the input nodes. The characteristics of MLPs include multiple layers and nonlinear activation for nodes of hidden and output layers, which distinguish MLPs from a linear perceptron and enable them to deal with nonlinear data.

The supervised classifiers mentioned above can be grouped into mainly two categories: linear and nonlinear classifiers. Linear classifiers aim to find a linear combination of features that separates classes of observations. However, such classifiers may not work well if a problem is nonlinear, in which data points cannot be separable with linear hyperplanes. Therefore, both types of classifiers, including LDA, SVMs, DT, RF, and MLP, were examined to identify an optimal classifier that performs well on inertial data for grading the quality of spastic movement. Leave-one-out cross-validation was employed to test classification performance. Each classifier was tested under four different conditions: common statistical features (FS1) derived from DS1 and DS2, and extra features (FS2) derived from both DS1 and DS2. Determining the severity of spasticity, as rated by the MAS, was considered a multiclass classification in this study, as each segment of the IMU signals was labeled with six classes. The accuracy of the classification was determined by applying Equation (5):

(5) $$\mathrm{Accuracy}=\frac{1}{N}\sum_{k=1}^{\left|G\right|}\sum_{x:g\left(x\right)=k}I\left(g\left(x\right)=\hat{g}\left(x\right)\right),$$

where *I* is the function that returns ‘1’ if the classes match and ‘0’ otherwise. Median accuracies were obtained for the different conditions. Moreover, statistical analyses were conducted to investigate the impact of feature type, segmentation technique, and machine-learning algorithm on classification performance. The Wilcoxon signed-rank test was used to compare the associated classification accuracies. The significance level (α) was set at 5%.

## 3. Results

Spastic movements rated by a rehabilitation therapist using the MAS and the number of data samples according to the segmentation techniques are reported in Table 4. The majority of participants had either no signs of spasticity in their elbow (35.4%) or minimal symptoms (27.1%). There was only one participant who showed the most severe degree of spasticity.

**Figure 4:**
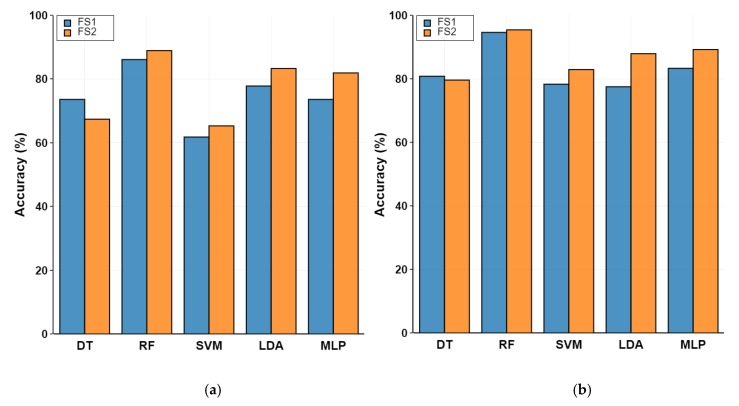
Classification results of classifiers depending on the two feature sets (FS1 and FS2) with the datasets prepared by applying the two different segment approaches: (**a**) DS1 and (**b**) DS2.

Classification performance was tested using a combination of datasets (DS1 and DS2) with segmentation and feature sets (FS1 and FS2) as shown in Figure 4.

When DS1 was used for classification, the median accuracy was 75.7%. As shown in Figure 4a, the median classification accuracies were 73.6% for FS1 and 81.9% for FS2 with DS1. The median value of accuracy increased by 8.3% when an extra 16 features were added to the common statistical features. However, the difference was not statistically significant, as confirmed by the results from a Wilcoxon signed-rank test (Z = −0.944, p = 0.345). The extra features did not have a significant influence on classification performance. Figure 4b summarizes the classification results, based on the features derived from the data with different segmentation techniques (DS2). The medians of classification accuracy for FS1 and FS2 were 80.8% and 87.9% respectively.

Table 5 summarizes the classification performance obtained from FS1 and FS2, regardless of the segmentation technique. The extra features improved classification by 5%. Although the extra 16 features (root mean square, mean, standard deviation, energy, spectral energy, absolute difference, variance extracted from pitch and roll, and two additional features: SMA and SV) added to the common statistical features (derived from both datasets DS1 and DS2) performed better, there was no significant increase in classification accuracy (Z = −1.784, p = 0.074).

Median accuracies were compared with regard to the segmentation technique. The performance increased by 7.4% when testing features were computed from DS2 (Table 6). Furthermore, the performance difference between segmentation techniques was statistically significant (Z = −2.701, p = 0.007). This indicates that having a data set segmented with 50% overlap had a significant positive impact on the classification accuracy.

The performance was compared in terms of classifier types. As summarized in Table 7, the most accurate classifier in this context was RFs, at nearly 95.4% accuracy (91.8% median accuracy), regardless of the type of segment technique applied or the number of features used. This was followed by MLPs and LDA, which classified the severity of spastic movement with approximately 80% accuracy. Our results indicate that SVMs were the least powerful classifier in this study.

Finally, the precision and recall for the best accuracy obtained with RFs using FS2 from DS2 are reported in Table 8. The classifier worked well overall, with the accuracy ranging from 92% to 100%. However, RFs showed relatively poor performance for discriminating MAS grade 1 and 1 +. MAS score 4 was perfectly classified, although there was only one participant with a MAS score of 4. Perfect classification was also observed for a MAS score 3.

## 4. Discussion

This study investigated whether grading of the degree of spasticity could be achieved by utilizing machine-learning algorithms and inertial signals collected during passive stretching. In previous studies, spasticity has been evaluated based on data collected from various types of sensors, including EMG, rotary angle sensors, load cells, force sensors, and IMUs [14,15,16,17,28]. However, such studies have aimed to provide such information for clinicians or therapists to improve the reliability of spasticity assessment. In addition, having to use multiple sensors requires sophisticated protocols and skills for sensor placement, e.g., preparing skin by removing hair and cleansing with alcohol before attaching EMG electrodes on the surface of target muscles. This kind of approach is generally employed in scientific studies. However, the ultimate purpose of the proposed method presented was to provide clients with a means of monitoring their own health-related status, especially in remote areas where healthcare professionals are unavailable. To accommodate this, a wearable device equipped with a minimum number of sensors was used to collect the signals reflecting characteristics of spastic movements. Specifically, spastic characteristics of the elbow were captured using a wearable device with IMU sensors; inertial sensors have been widely used in similar applications, such as outcome assessment in healthcare domain [23,26,29,49,50].

Our proposed method considers spasticity assessment as a classification problem, in contrast to a previous study [29]. This is because that there is some controversy over whether to use an ordinal or a categorical scale, due to the addition of the 1 + score. It has been argued that the relationship between 1 and 1 + is hierarchical [51]. In addition, there iso no unique definition of zero on this scale, in contrast to the original Ashworth scale; thus, there is little information on whether the distances between 1 and 1 + and 1 + and 2 are equal [52]. Due to insufficient evidence for using an ordinal scale, the proposed method grades the degree of spasticity using the MAS as a classification problem.

Within the proposed method, several approaches were applied to identify the optimal combination of features, segmentation methods, and supervised learning classifiers that perform classification of MAS scores with confidence. In terms of segmentation methods, a comparison of two segmentation techniques indicated that allowing a 50% overlap between previous and subsequent signals was a major factor that impacted classification performance significantly. This is consistent with current findings [34,53,54] that have found that the accuracy increases systematically with segmentation methods that allow overlap. Segmentation without overlap loses some information between previous and next segments, which results in a dataset with poor representation. On the other hand, increased performance could be due to not only better representation captured by segmentation with overlap but also a bigger number of datasets available for training machine-learning algorithms.

Feature-wise comparison revealed that FS1 (the commonly used statistical features) was as effective as FS2 (those combined with extra features computed from pitch and roll representation of inertial signals) due to the fact that such increases in classification performance were not enough to be statistically significant. Although FS2 caused rather systematic increases in accuracy for most of the classifiers tested, it imposed a heavier computational burden in comparison to FS1. Therefore, advantages of using the extra features is not clear; the tradeoff among accuracy, response time, and battery run-time should be considered carefully and will likely depend on the priorities of application.

In the proposed method, the key priority was to provide both healthcare professionals and nonprofessionals with clinical indicators, so that they could rate the degree of elbow spasticity in patients by discriminating the different levels indicated by inertial sensors. While other studies have derived biomarkers from EMG, force, angle, and inertial data (e.g., resistance, angle, angular acceleration, and velocity [27,29]), the proposed method utilizes different types of features that are widely used in human activity recognition [53]; the performance results were high, indicating that our approach is feasible.

In terms of classifier performance, statistical analyses confirmed that the machine-learning classifiers tested had no significant influences. Nevertheless, nonlinear classifiers tended to work better than linear ones. In fact, RFs outperformed LDA, SVMs, and MLPs. In addition, MLPs performed relative better than the linear classifiers, with the exception that LDA only outperformed MLP when DS2 was tested. Our findings are in accordance with those of previous studies [27,53].

There were a few data samples that represented MAS 3 and 4. However, they were perfectly separated, indicating that the characteristics of MAS 3 and 4 are clearer than other MAS scores according to precision and recall metrics. On the other hand, relatively lower subset accuracy was observed with MAS 1 and 1 +. This is a known issue associated with the MAS. The initial version of Ashworth scale was modified with an addition score 1 +, to discriminate from MAS score 1, as it represents a state that indicates resistance through less than half of the movement. This was introduced to increase the sensitivity of the scale. However, the additional level of measurement (1 +) may have caused poorer agreement between raters, leading to lower reliability than the original Ashworth scale [51]. The ambiguity of scoring the degree of spasticity may also have led to poorer quality for labeling data for classification. Further investigation with regards to the 1 + point of the MAS is beyond the scope of this study.

The highest classification accuracy of MAS score was achieved by RFs in combination with 50% overlapping for segmentation and a total 58 features. Our findings confirm that the approach proposed is acceptable. Besides, it only requires a wearable device equipped with IMUs, which is the most frequently used sensors for fitness trackers or smart watches. The simplicity of the proposed method is what makes it possible to incorporate the method into tele-rehabilitation applications that deliver rehabilitation services—including rehabilitation interventions [55], clinical assessments [56], and consultations [57]—over tele-communication networks as well as the Internet [58].

The empirical results reported herein should be considered in the light of some limitations. Increased segmentation overlap leads to higher accuracy in most cases [53]. However, different percentages for segment overlaps were not examined. It would be worth investigating the impact of the amount of segmentation overlap on classification performance. In addition, the proposed method requires features to be computed from data, as the method employs classic supervised machine-learning classifiers. Recent advances in machine learning, including deep learning, are rapid; a considerable number of studies have reported that deep-learning methods outperform classic machine-learning algorithms. Therefore, it is a reasonable next step to examine deep-learning models and investigate factors potentially beneficial to performance improvement. Although high classification performance was achieved across various conditions tested, it does not guarantee that our method would perform well on a larger dataset due to the limited sample size, e.g., MAS score 3 and 4. Having more data would help the model be more accurate and generalizable. We expect to address such issues by examining larger sample sizes and applying deep learning to enhance the current work.

## 5. Conclusions

This study investigated the use of inertial data for evaluating the severity of elbow spasticity using machine-learning algorithms. The findings confirm that careful processing of inertial data collected during spasticity assessment using MAS-enabled machine-learning classifiers can be used to determine the severity of spasticity (six degrees, ranging from 0 to 4 including 1 +). This work not only successfully demonstrates how wearable technology and machine learning can be used to generate a clinically meaningful index, but also proposes a method that offers rehabilitation patients an opportunity to monitor the degree of spasticity, even in places where the direct help of therapists is inaccessible.

## Figures and Tables

**Table 1:** Demographic information of study participants.

Characteristics	Male	Female
No. of participants	26	22
Age (mean ± std)	61.2 ± 13.7	77.8 ± 10.1
Diagnosis (CVA/SCI)	24/2	21/1
Affected side (none/right/left)	9/7/10	8/10/4

CVA: cerebrovascular accident; SCI: spinal cord injury.

**Table 2:** MAS scoring description and corresponding labels of the MAS scores used for supervised learning.

Scores	Label	Description
0	0	No increase in muscle tone
1	1	Slight increase in muscle tone, manifested by a catch and release, or by minimal resistance at the end of the range of motion when the affected part(s) is moved in flexion or extension
1 +	2	Slight increase in muscle tone, manifested by a catch, followed by minimal resistance throughout the remainder (less than half) of the ROM
2	3	More marked increase in muscle tone through most of ROM, but affected part(s) easily moved
3	4	Considerable increase in muscle tone, passive movement difficult
4	5	Affected part(s) rigid in flexion and extension

ROM: range of motion.

**Table 3:** Description of feature sets.

	Acceleration from 3-Axis (*x*, *y*, *z*)	Angular Velocity from 3-Axis (*x*, *y*, *z*)	Roll	Pitch	Additional Features
FS1 (n = 42)	root mean square, mean, standard deviation, energy, spectral energy, absolute difference, variance		-	-	-
FS2 (n = 58)	root mean square, mean, standard deviation, energy, spectral energy, absolute difference, variance				SMA, SV

**Table 4:** Results of MAS obtained with study participants recruited (n = 48) and number of data samples segmented by two different techniques.

Range of MAS		0	1	1 +	2	3	4	Total
Number of participants		17	13	7	6	4	1	48
Dataset	DS1 (nonoverlapping)	51	39	21	18	12	3	144
	DS2 (50% overlapping)	85	65	35	30	20	5	240

**Table 5:** Median classification accuracy according to the number of features: common statistical features (n = 42) and the common features with the extra 16 features (n = 58).

Number of Features	FS1	FS2
Median Accuracy	78.1%	83.1%

**Table 6:** Median classification accuracy according to the segmentation technique: data segmented without overlapping (DS1) and data segmented with 50% overlapping (DS2).

Dataset	DS1	DS2
Median Accuracy	75.7%	83.1%

**Table 7:** Median classification accuracy according to the machine learning classifiers tested regardless of segmentation technique.

Classifiers	DT	RF	SVM	LDA	MLP
Median Accuracy	76.6%	91.8%	71.8%	80.6%	82.6%

**Table 8:** Precision and recall of RF with FS2 derived from DS2.

MAS scores	Precision	Recall	Accuracy
0	98%	98%	98%
1	90%	94%	92%
1 +	97%	89%	93%
2	97%	97%	97%
3	100%	100%	100%
4	100%	100%	100%
